# A Comprehensive Assessment of Tear-Film-Oriented Diagnosis (TFOD) in a Dacryoadenectomy Dry Eye Model

**DOI:** 10.3390/ijms242216510

**Published:** 2023-11-20

**Authors:** Saki Sakakura, Emi Inagaki, Yuichiro Ochiai, Masatoshi Yamamoto, Naofumi Takai, Taeko Nagata, Kazunari Higa, Yasunori Sato, Hiroshi Toshida, Dogru Murat, Masatoshi Hirayama, Yoko Ogawa, Kazuno Negishi, Shigeto Shimmura

**Affiliations:** 1 Department of Ophthalmology, Keio University School of Medicine, 35 Shinanomachi, Shinjuku-ku, Tokyo 160-8582, Japanmar_hirayama@keio.jp (M.H.); kazunonegishi@keio.jp (K.N.); 2 Department of Physiology, Keio University School of Medicine, 35 Shinanomachi, Shinjuku-ku, Tokyo 160-8582, Japan; 3 Japan Society for the Promotion of Science, 5-3-1 Kojimachi, Chiyoda-ku, Tokyo 102-0083, Japan; 4 Kitayama Labes Co., Ltd., 3052-1 Arai, Ina City 396-0025, Japan; 5 Cornea Center and Eye Bank, Tokyo Dental College Ichikawa General Hospital, 5-11-13 Sugano, Ichikawa 272-8513, Japan; higakazunari@tdc.ac.jp; 6 Department of Preventive Medicine and Public Health, Keio University School of Medicine, 35 Shinanomachi, Shinjuku-ku, Tokyo 160-8582, Japan; yasunori.sato@keio.jp; 7 Department of Ophthalmology, Juntendo University Shizuoka Hospital, Nagaoka 1129, Izunokuni City 410-2295, Japan; toshida@juntendo.ac.jp; 8 Department of Ophthalmology, Tsurumi University, 2-1-3 Tsurumi, Yokohama 230-0063, Japan; 9 Department of Clinical Regenerative Medicine, Fujita Medical Innovation Center, Fujita Health University, Haneda Innovation City Zone A, 1-1-4 Hanedakuko, Ota-ku, Tokyo 144-0041, Japan

**Keywords:** dry eye, tear film breakup, animal model, dry eye therapy

## Abstract

Tear film instability is a major cause of dry eye disease. In order to treat patients with short tear film breakup time (TBUT)-type dry eye, the development of tear film stabilizing agents is essential. However, the lack of an appropriate animal model of tear film instability has made drug development difficult. Although rabbit dry eye models have been reported in the past, there are only a few reports that focus on tear film instability. Herein, we assessed the tear film stability of a rabbit dry eye model induced by dacryoadenectomy. A clinical evaluation of the ocular surface, interferometry, and histological assessments of the cornea and conjunctiva were performed. Following the removal of the lacrimal glands, TBUT was shortened significantly, with dimple and random breakup patterns prominently observed. Furthermore, the blink rate in this model increased after dacryoadenectomy, suggesting that this model partially captured the phenotypes of human short TBUT-type dry eye and may be useful as an animal model for investigating potential drug candidates.

## 1. Introduction

Dry eye is one of the most common ocular diseases worldwide [1,2,3,4,5]. While deficiency in tear production was originally regarded as the pathophysiology of dry eye, several reports have suggested that the instability of the tear film may be the primary etiology of the disease [6,7,8,9,10,11]. The concept of tear-film-oriented diagnosis (TFOD) [12] was proposed to allow for a more logical approach to therapy. From an epidemiologic perspective, short tear film breakup time (TBUT)-type dry eye due to tear film instability is reported to be more common than other types of dry eye, such as aqueous tear deficiency [13,14]. Even though patients with short TBUT-type dry eye suffer from the same symptoms as patients with aqueous-tear-deficient dry eye [15], the affected layers of lacrimal fluid and proper treatments are different among the subtypes. In addition, TBUT is a major diagnostic criterion for dry eye [6,8,9,11,12]. Thus, the establishment of a short TBUT-type dry eye model that can be used for a TFOD-based approach to therapy is necessary for the development of new drugs. 

Several dry eye models have been reported in the literature [16,17,18]. In particular, rabbit models are often used due to several advantages over mice or rats. First, the size of rabbit corneas is approximately 15 mm in diameter [19], which will allow for the detailed examination of the ocular surface. Second, many eye drops contain ester groups to enhance drug permeability. However, while carboxylesterases exist in humans and rabbits, mice do not express the enzyme in tears [20,21]. Carboxylesterases are involved in the bioactivation of several eyedrops following application to the ocular surface [22]. Among subtypes of carboxylesterases, carboxylesterase 1 is an active factor in human eyes, which is similar to rabbits [20,22]. The evaluation of this enzyme in the cornea may be important for local drug delivery [22]. Due to such similarities with humans, rabbits are suitable for translational research [23]. Multiple approaches to induce dry eye in rabbits have been reported, including chemical [24,25], surgical [26,27,28,29], and environmental [30] interventions. Dacryoadenectomy has been used in many studies investigating tear volume and the consequences of tear volume reduction, although the results have been inconsistent [27,28,29,31]. 

The need to better understand the pathology of dry eye has led to the development of new strategies for evaluating the ocular surface and tear dynamics in detail [32,33]. The Ocular Surface Analyzer (OSA) (SBM Sistemi S.r.l., Torino, Italy) is an instrument that was developed to diagnose dry eye from various perspectives, including tear meniscus height (TMH), lipid layer thickness (LLT) using interferometry, and meibography [34,35,36]. This is a non-invasive diagnostic tool allowing for the assessment of tear dynamics under natural conditions.

In this study, we established an animal model similar to human dry eye by using OSA in combination with dacryoadenectomy in rabbits to perform ocular assessments in alignment with the TFOD approach.

## 2. Results

### 2.1. Tear Film Breakup Time (TBUT) and Breakup Patterns

**Figure 1:**
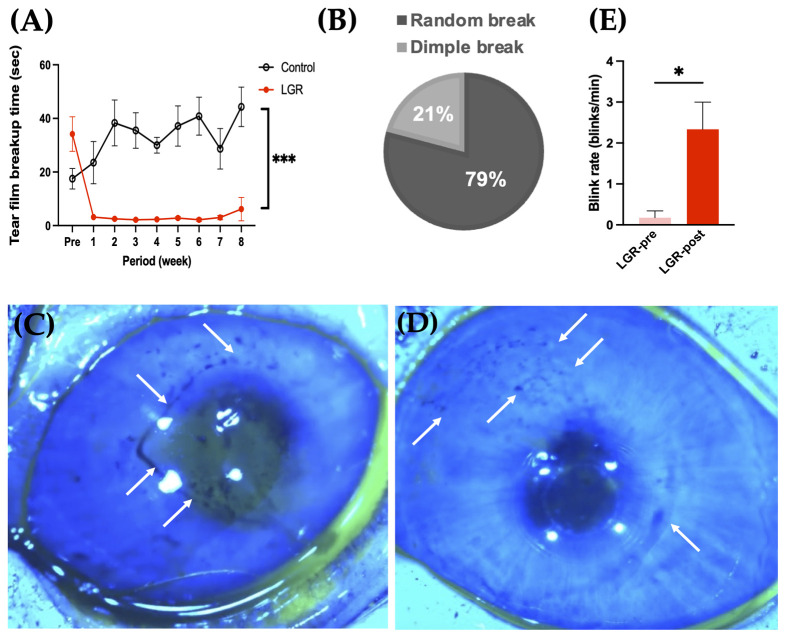
Time course and pattern of tear film breakup time (TBUT). (**A**) Comparison of TBUT between control and lacrimal gland removal (LGR) eyes at different times (*n* = 6, *** *p* < 0.001). (**B**) The ratio of breakup patterns in LGR eyes. (Six rabbits, a total of 48 eyes in each group, were analyzed from one week after LG removal to the end of the study at eight weeks). (**C**,**D**) Breakup pattern of LGR eyes: (**C**) dimple breakup pattern and (**D**) random breakup pattern. Arrows indicate breakup. (**E**) Blink rate (blinks/min) (*n* = 6, * *p* = 0.01).

One week after lacrimal gland removal (LGR), the TBUT of LGR eyes (2.9 ± 0.5 s) decreased compared with contra-lateral control eyes (23.5 ± 7.2 s). The TBUT of LGR eyes continued to be significantly shorter until the end of the study at eight weeks (*p* < 0.001) (Figure 1A). 

We next assessed tear film breakup patterns to classify the subtypes of dry eye. In the LGR eye, a random break was seen commonly (79%, *n* = 38), and a dimple break was also observed (21%, *n* = 10) (Figure 1B). The variation of breakup patterns was similar during all observation periods. On the other hand, area breakup and line breakup, which result from aqueous tear volume reduction, were not detected in this model (Figure 1B). A stripe-shaped dimple breakup was observed soon after lifting of the upper eyelid as the tear film lipid layer passed over the central cornea (Figure 1C), suggesting the impaired wettability of the corneal surface. A random pattern is defined as a random-shaped breakup when eyes are held open (Figure 1D), suggesting evaporative dry eye. The blink rate in LGR eyes was 0.2 ± 0.2 blinks/min pre-treatment, which increased to 2.3 ± 0.7 blinks/min after dacryoadenectomy (*p* = 0.01) (Figure 1E).

### 2.2. Corneal Fluorescein Staining Scores

**Figure 2:**
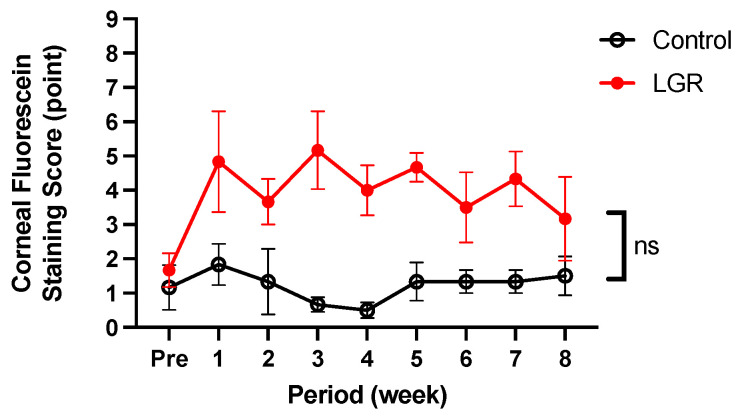
Time course of corneal fluorescein score. There were no significant differences (ns) between control and lacrimal gland removal (LGR) eyes at every experimental period (*n* = 6).

The corneal fluorescein staining score at baseline was 1.7 ± 0.1 in LGR eyes and 1.2 ± 0.6 in control eyes. LGR resulted in higher scores than the control, but there was no significant difference between the two groups overall (*p* = 0.15). Eight weeks after LGR, the corneal fluorescein staining score was 3.2 ± 0.2 in LGR eyes and 1.5 ± 0.5 in control eyes (Figure 2). 

### 2.3. Schirmer’s I Test and Tear Meniscus Height (TMH)

**Figure 3:**
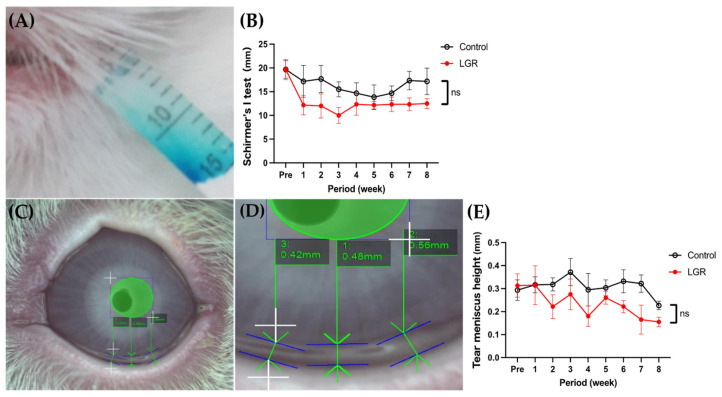
(**A**,**B**) Schirmer’s I test procedure (**A**) and its time course of control and lacrimal gland removal (LGR) eyes (**B**) (*n* = 6). (**C**–**E**) Tear meniscus height (TMH) measurement procedure with OSA: (**D**) high-power field of (**C**), and (**E**) time course of control and LGR eyes (*n* = 6). No significant difference (ns) was detected between control and lacrimal gland removal (LGR) eyes.

The Schirmer’s I test was performed to quantify aqueous tear production (Figure 3A). At baseline, the tear volume of LGR and control eyes was 19.7 ± 1.9 mm and 19.7 ± 1.7 mm, respectively. LGR resulted in a lower tear volume than the control, but no significant difference was detected at any time point between the LGR and control groups (*p* = 0.78). Eight weeks after the procedure, the tear volume of LGR was 12.5 ± 1.0 mm, while that of the control was 17.2 ± 2.5 mm (Figure 3B). Regarding TMH, the distance between the cornea–meniscus junction and the lower eyelid–meniscus junction was measured using the OSA and was assessed (Figure 3C,D). Prior to the procedure, the TMH of LGR and control eyes was 0.33 ± 0.04 mm and 0.29 ± 0.05 mm, respectively. LGR resulted in lower TMH than the control, but there was no significant difference at any time point between the LGR and control groups (*p* = 0.57). Eight weeks after the procedure, the TMH of LGR was 0.16 ± 0.02 mm, while that of the control was 0.23 ± 0.02 mm (Figure 3E).

### 2.4. Lipid Layer Thickness (LLT) Grading Using Interferometry

**Figure 4:**
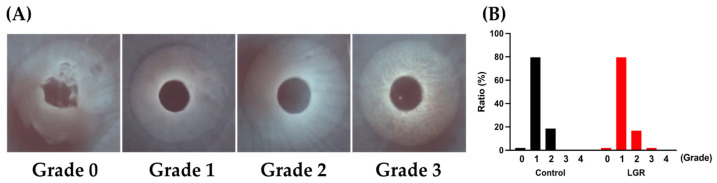
(**A**) Typical images of grading scales using interferometry in rabbits. (**B**) Results of the ratio of interferometry grading between control and lacrimal gland removal (LGR) eyes. There were no significant differences. (Six rabbits, a total of 54 eyes in each group, were analyzed from pre-treatment to the end of the study at eight weeks).

The assessment of LLT using interferometry is an essential aspect of TFOD, and the phenomenon of interference fringes enables us to measure the thickness of the tear film lipid layer. Among five grades of LLT, grades 0–3 were observed in this study (Figure 4A), and the most frequent grade was grade 1 (Figure 4B). The distribution of grading scores was similar between LGR and control groups (Figure 4B).

### 2.5. Histological Assessment

**Figure 5:**
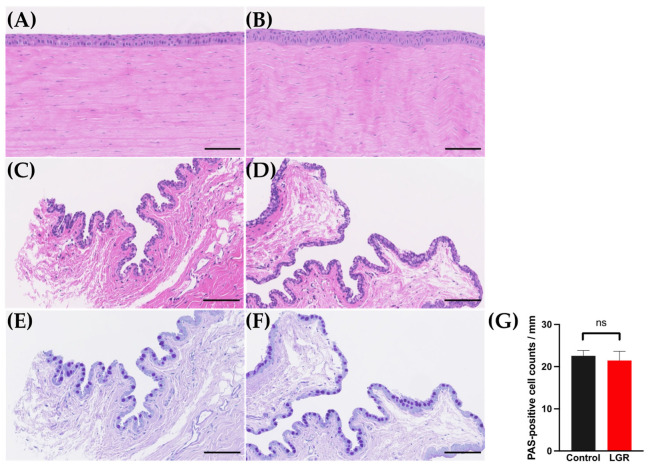
Histological assessment of cornea and conjunctiva. Scale bars are 100 μm. (**A**,**B**) Hematoxylin-eosin (HE) staining of the control (**A**) and lacrimal gland removal (LGR) cornea (**B**). (**C**,**D**) HE staining of the control (**C**) and LGR conjunctiva (**D**). (**E**,**F**) PAS staining of the control (**E**) and LGR conjunctiva (**F**). (**G**) The results of PAS-positive cell counts (/mm) in the control and LGR conjunctiva. There was no significant difference (ns) (*n* = 6).

Eight weeks after LG removal, three to five layers of corneal epithelial cells were observed in both the control group and the LGR group. There was no thinning of the epithelial layer or stromal edema in either group (Figure 5A,B). 

The histological assessment of the bulbar conjunctiva showed a cuboidal epithelial layer in both the control and the LGR group. There was no difference in the thickness of the conjunctival epithelium (Figure 5C,D). The number of PAS-positive goblet cells, which included a high percentage of mucin, were 21.4 ± 2.2 cells/mm in the LGR group and 22.5 ± 1.2 cells/mm in the control group (*p* = 0.67) (Figure 5E–G).

## 3. Discussion

In this study, we evaluated the stability of tear fluid on the ocular surface using dry eye model rabbits. Although the effect on tear volume via dacryoadenectomy was limited, the consistent shortening of TBUT throughout the experiment suggests that this model may be advantageous for new drug development studies.

Rabbit models of dry eye have often been used as models because of their similarity to human dry eye [21]. However, the induction of dry eye through surgical dacryoadenectomy resulted in inconsistent dry eye phenotypes regarding tear volume reduction [23]. Rabbits with lacrimal gland extirpation were referred to as “mild” dry eye models regarding tear volume reduction [29]. Therefore, we assessed the effect of LG removal in rabbits using TFOD methods and compared the similarities with human dry eye. 

Our LG removal model exhibited signs of short TBUT-type dry eye: decreased TBUT, dimple, random breakup pattern, and a slightly higher corneal fluorescein staining score. TBUT values of 18–60 s are considered normal in rabbits [23]. From 1 to 8 weeks after LG removal, the TBUT value continued to be significantly lower than the control. Among previous reports in which dacryoadenectomy was performed, Honkanen [27] reported that TBUT was 4.5 ± 1.2 s, which is compatible with our results. No other reports involving dacryoadenectomy performed breakup time measurements [28,29,31,37]. According to a report from the Tear Film and Ocular Surface Society Dry Eye Workshop II (TFOS DEWS II) [38], there is a lack of studies focused on tear film instability using dry eye models. Our method with TBUT measurements using the OSA, Schirmer’s I test, and histological examination is in accordance with the TFOS DEWS II Diagnostic Methodology report [32], as well as the TFOS DEWS II pathophysiology report [10]. One advantage of this study is that the phenotype of BUT shortening observed in the intervention group can be reproduced stably up to 8 weeks. Blinking is also related to tear film dynamics [39] and is both a cause and an effect of dry eye [40]. In our model, the blink rate increased in LGR eyes. This result was compatible with the fact that a short blink interval is a characteristic of dry eye patients [41]. However, blinking is subject to multiple factors such as ocular surface conditions, muscular fatigue, and psychological factors [42]. To further validate our results, a comparison with sham-operated groups may be necessary. 

To our knowledge, this report is the first to consider tear film breakup patterns in rabbits. Dimple and random patterns suggested that this model imitated short TBUT-type dry eye in humans. The most common breakup pattern in LGR eyes was the random pattern, which could also be seen in normal eyes [12]. As the normal rabbit blink rate is as low as one to three times per minute [23], it is possible that random patterns could be detected most often in rabbits. This low blink rate makes objective TBUT measurement by examiners difficult, especially in normal rabbits. Thus, automatic assessments using the OSA should ensure more consistent results. The OSA also enables us to judge breakup patterns and dry-eye-associated findings such as changes in the tear film lipid layer non-invasively. The detailed mechanism of TBUT reduction in post-dacryoadenectomy rabbits has not been elucidated [18,43]. LG produces mucins including MUC1, MUC4, MUC5AC, MUC5B, MUC7, and MUC19 [44,45]. Although conjunctival goblet cells are the primary source of the main gel-forming mucin MUC5AC, the number of goblet cells was not significantly different between LGR and control groups in our study. The removal of the main lacrimal gland may change the balance between gel-forming and membrane-associated mucins. In order to further characterize this model, the investigation of ocular surface MUC5AC, MUC1, MUC4, and MUC16 expression is necessary [46]. In addition, the further assessment of goblet cells using conjunctival impression cytology may allow follow up on changes over time. Combining LGR with diquafosol sodium as a positive control [47] may also help validate this model. Future studies testing the effectiveness of dry eye treatments using this model would be valuable for pre-clinical studies. Further investigations will explore the impact of removing the lacrimal gland on the composition of tears through the integration of molecular biological and proteomic studies.

In our model, no significant differences were detected in Schirmer’s I test, TMH, tear film LLT grading, or the histology of cornea and conjunctiva, suggesting that this model does not show all typical signs of aqueous-tear-deficient dry eye. Normal Schirmer’s I test is reportedly 13–30 mm in rabbits [23], and the average in our LGR model was 13 mm, which is within the normal range. Normal TMH is reported to be 0.2–0.5 mm with the OSA [48] and 0.2 mm with FD-OCT [49]. LG removal in our model did not result in significant aqueous tear volume reduction. The results of the Schirmer’s I test after dacryoadenectomy in previous reports were inconsistent. In some reports, the tear volume decreased significantly to 9–12 mm [27,29,31], while in other reports, it was approximately 20 mm [28,37]. One of the reasons for this inconsistency may be attributed to the orbital lobe of the superior LG, which is located in the zygomatic bone and cannot be accessed through a periorbital approach [27]. Removal of the superior LG in the zygomatic bone was described by Honkanen et al. by using X-ray images with contrast agents [27]. While tear volume reduction may be possible with this approach, the partial removal of the LG as in our study is sufficient to study short BUT-type dry eye.

The tear film lipid layer inhibits tear breakup since lipid elasticity prevents aqueous layer thinning [50]. A previous report revealed that the thinning of the tear film lipid layer was related to aqueous-tear-deficient dry eye and meibomian gland dysfunction [36,51,52]. The assessment of the tear film lipid layer using interferometry helps to distinguish the subtypes of dry eye [53]. A previous report on rabbit interferometry showed that the most frequently observed grade in normal female rabbits was grade 1 [48], which is similar to our study. Previous reports proposed that higher grades, especially grades 3, 4, and 5, suggested aqueous-tear-deficient dry eye in humans [54,55]. No noticeable difference in interferometry pattern was detected between LGR and control eyes in this study, suggesting that the phenotype of severe aqueous tear deficiency was not detected in our LGR model. However, the normal tear film lipid layer grade in rabbits ranged from one to four [48], suggesting that applying this evaluation method to rabbits may be difficult. Furthermore, rabbit meibomian glands and Harderian glands have a unique family of lipids which are not detected in humans [56,57]. Furthermore, while gel-forming mucin MUC5AC [47] and dry-eye-related antimicrobial proteins such as lactoferrin, lipocalin, and lipophilin are also identified in rabbit tear [58,59,60], lysosome is minimally identified [59]. This feature could also make comparing the tear film lipid layer between rabbits and humans difficult.

Utilizing a suitable animal model for assessing potential new drugs is important, especially for diseases with a specific etiology, such as tear film stability in the case of short TBUT-type dry eye [61]. Ocular assessments using the TFOD approach revealed that the LGR rabbit model exhibited signs of short TBUT-type dry eye, which is the prevalent dry eye type in humans. This model should prove useful for future studies on the pathophysiology of dry eye and drug development.

## 4. Materials and Methods

### 4.1. Animals and Ethics Statement

Female New Zealand white rabbits (*n* = 6, 12 eyes) were reared under standard laboratory conditions (22 ± 3 °C, 12 h light–dark cycle) at Kitayama Labes (Nagano, Japan). The mean age was 13 weeks, and the weight ranged from 2.0 kg to 2.5 kg. Animals had free access to food and water. No ocular abnormalities were detected in any of the animals. All animal experiments were performed in accordance with the ARVO Statement for the Use of Animals in Ophthalmic and Vision Research. The animal study protocol was approved by the Institutional Review Board of Kitayama Labes (IBC-59-044).

### 4.2. Removal of Lacrimal Glands and Nictitating Membranes

**Figure 6:**
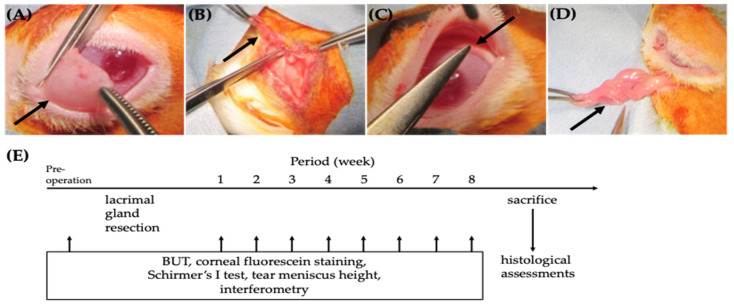
(**A**–**D**) Surgical procedures: removal of the nictitating membrane (**A**, black arrow), orbital lobe of the superior lacrimal gland (LG) (**B**, black arrow), palpebral lobe of superior LG (**C**, black arrow), and inferior LG (**D**, black arrow). (**E**) Time course of ocular surface evaluations.

Dry eye models were induced by removing the superior and inferior lacrimal glands (SLG, ILG) and nictitating membrane (NM) of the left eye. Operative procedures were carried out under general anesthesia with medetomidine hydrochloride (0.5 mg/kg, Nippon Zenyaku Kogyo Co., Ltd., Fukushima, Japan), midazolam (2 mg/kg, Maruishi Pharmaceutical Co., Ltd., Osaka, Japan), and butorphanol (0.5 mg/kg, Meiji Seika Pharma Co., Ltd., Tokyo, Japan). The skin of the surgical area was shaved, and the surgical site was disinfected with 10% Povidone-Iodine (Mundipharma K.K., Tokyo, Japan). For the left NM removal, oxybuprocaine hydrochloride 0.4% (Santen Pharmaceutical Co., Ltd., Osaka, Japan) was applied to the left eye. The NM was pulled out in front of the cornea, and xylocaine 1% (0.3 mL/head, Sandoz Pharma K.K., Tokyo, Japan) was injected into the NM. Five minutes later, the NM was excised at the base (Figure 6A). For LG removal, a curve-shaped incision was made along inferior and lateral orbital rims. After the dissection of supporting tissues, the orbital lobe of superior LG (Figure 6B), palpebral lobe of superior LG (Figure 6C), and inferior LG (Figure 6D) were removed, respectively. After the removal of LGs, the muscle layer was closed with 5-0 Coated Vicryl sutures, and skin was closed with 3-0 Ethilon Nylon sutures. Bupivacaine 0.5% (0.5 mL at maximum, Sandoz Pharma K.K., Tokyo, Japan) was injected subcutaneously for perioperative pain control. Oxytetracycline hydrochloride and hydrocortisone ointment (Yoshindo Inc., Toyama, Japan) were applied to the surgical wounds and oxytetracycline hydrochloride/polymyxin B sulfate (30 mg/kg, Zoetis Japan Inc., Tokyo, Japan) was administered intramuscularly.

### 4.3. Ocular Surface Evaluation

All examinations except Schirmer’s I test were performed under general anesthesia. First, Schirmer’s I test was performed. Then, interferometrical analysis and tear meniscus assessment were performed with an Integrated Clinical Platform Ocular Surface Analyzer for Veterinary Use (OSA-Vet). TBUT measurement and corneal fluorescein staining assessment followed. All rabbits were examined weekly before and after SLG, ILG, and NM excision for eight weeks. After completing evaluations, all rabbits were sacrificed, and histological assessments were performed (Figure 6E).

### 4.4. Tear Film Breakup Time (TBUT) and Breakup Pattern Assessment, Blink Rate, and Corneal Fluorescein Staining

After eyes were instilled with fluorescein, manual blinking was performed several times. Video images of the cornea were taken with OSA-Vet for 60 s, and TBUT values were judged using the OSA-Vet automatically. If the breakup was not observed by 60 s, TBUT was recorded as 60 s. Videos were observed by two ophthalmologists (SS, EI) who assessed breakup patterns. There are five main patterns of corneal fluorescein breakup: dimple, spot, random, area, and line breakup, with each pattern reflecting the instability of tear film layers due to different causes [12]. Briefly, in dimple and spot breakup, impaired wettability of the corneal surface causes tear breakup, and the degree of corneal epithelial damage or tear volume reduction is reported to be mild [12]. A random pattern suggests evaporative dry eye, while area and line breakup suggest aqueous tear deficiency [12]. Blink rate was counted from a video recording of rabbits by two ophthalmologists (SS, EI). Videos were captured at the time of pre-treatment and one week after the surgery.

For corneal fluorescein scoring, cornea images were divided into three areas horizontally, and the extent of fluorescein staining was graded on a scale of 0 to 3 [62]. The total score (0–9) was counted.

### 4.5. Schirmer’s I Test

Under natural conditions, rabbits were stabilized with restraining devices. A Schirmer paper strip (Katena Products Inc., Parsippany, NJ, USA) was inserted into the lower lateral conjunctival sac. Inserting a paper strip into this area seems to cause minimal pain to rabbits, and therefore we conducted Schirmer’s I test without local anesthesia to evaluate natural tear volume [62]. In case the insertion caused an uncomfortable sensation for rabbits, we reassessed the tear volume after a certain period of time. The length of moistened papers was measured after 5 min. 

### 4.6. Lipid Layer Thickness (LLT) Assessment using Interferometry

Videos were captured every week from pre-treatment to eight weeks using OSA-Vet. Videos were examined in a blind fashion by two ophthalmologists (SS, EI), and LLT was graded according to five frame-grading scales [63] as follows: absence of aqueous phase in grade 0 (0–15 nm of lipid layer), faintly visible meshwork pattern in grade 1 (15–30 nm), compact meshwork pattern with gray waves in grade 2 (31–60 nm), meshwork with some colorful waves in grade 3 (61–100 nm), waves with multiple colors in grade 4 (more than 100 nm).

### 4.7. Histological Assessments of Cornea and Conjunctiva

After all ocular evaluations were completed at eight weeks, rabbits were humanely euthanized with an intravenous injection of thiopental sodium (1 mL/kg, Nipro ES Pharma Co., Ltd., Osaka, Japan). The cornea and bulbar conjunctiva were resected and fixed in 10% formalin. Specimens were dehydrated, embedded in paraffin, cross-sectioned, and stained with hematoxylin-eosin (HE) or Periodic acid-Schiff (PAS). Samples were scanned with NanoZoomer-XR C12000 (Hamamatsu Photonics K.K., systems division, Shizuoka, Japan). The morphology of the cornea was observed under 20× magnification. Images of the whole bulbar conjunctiva were captured, and the total number of PAS-positive cells and the length of the conjunctiva in one eye were counted, respectively, with ImageJ version 1.53t [64]. The number of PAS-positive cells per 1 mm conjunctiva was calculated.

### 4.8. Statistical Analysis

Statistical analysis was conducted with GraphPad Prism 9.0 (GraphPad Software, Boston, MA, USA) and SAS Ver. 9.4 (SAS Institute Inc., Cary, NC, USA). After confirming normal distribution for all variables with Q-Q plot and Shapiro–Wilk test, statistical comparisons were analyzed using a generalized linear mixed model with fixed effects for time, group, and their interaction, using a compound symmetry covariance matrix. Comparison of the two groups was conducted using the unpaired *t*-test. Values represent the mean ± standard error (SE). For all analyses, differences were considered statistically significant when the two-sided *p* value was less than 0.05. The significance level was set at * *p* < 0.05, ** *p* < 0.01, and *** *p* < 0.001.

## Data Availability

All data are provided in the main text.
